# Account of an Individual Who Swallowed a Drachm of Powdered Cantharides

**Published:** 1825-12

**Authors:** James Torrie


### ART. IV.—*Account of an Individual who swallowed a Drachm of Powdered Cantharides.* By JAMES TORRIE, M.D.

I BEG leave to trouble you with the following account of a case which occurred to me some months ago; and, if you think it worthy of a place, you will confer a favour on me by inserting it in your valuable Journal. My reason for sending it to you is, that an erroneous account of the medical evidence given on the trial to which it gave rise having appeared in some of the newspapers, I am desirous, through what I conceive the proper medium, to give an accurate statement of the facts.

On the morning of the 2d of May last, I was called to visit George Middleton, a man of a full habit of body, about forty years of age. He complained of much heat and pain in the region of the stomach, which was in so irritable a state that it would retain nothing. His tongue, throat, and gums, were ulcerated. There was a copious discharge of saliva. He had a frequent inclination to make water, and great pain when he attempted to do so. He had a good deal of thirst, and a small pulse, above a hundred in the minute. On inquiring the cause of these symptoms, I was informed that, two days previously to my seeing him, he had been drinking with a neighbour, and had at that time got a dose of tincture of Spanish flies in a glass of mulled porter. Notwithstanding a positive assurance that *tincture* of cantharides had been given him, I felt some difficulty in believing that the state of his mouth, throat, and tongue, could have been produced by the tincture, diluted in the manner described by the patient and his friends.

As the more prominent symptoms, however, were such as might have been produced by an over-dose of cantharides in some shape or other, I felt no difficulty about the treatment. It would be needless to trouble you with a detailed account of the method of cure. It is sufficient to say that it consisted in giving diluents, opium, the camphorated mixture, and occasional doses of castor-oil.

On the day after I had first seen him, his symptoms continuing much the same, the Procurator Fiscal, who is the public prosecutor in every Scotch burgh, called on me to request that I would make out an account of the man's case, for the information of the lord advocate. This I promised to do on the following day; and, the man being then somewhat relieved, I drew up a report of the general features of the case, concluding it with a hope that all danger would soon be over.

I regret that I have not an exact copy of that report in my possession; but, to the best of my recollection, it contained nothing more than what I have stated to you concerning my visits to the patient, and the symptoms I observed.

On the fourth day, as Middleton still continued to improve, I was requested by the friends of those who, being implicated in the administration of the cantharides, had been sent to jail, to sign a certificate that the man who had taken it was out of danger, in order that the prisoners might be admitted to bail. This, however, I deferred doing till the following day, when the more alarming effects of the cantharides being in a great degree removed, and the stomach of the patient capable of retaining a little liquid nourishment, I gave the certificate in the usual form. I continued my attendance for some time longer, until the ulcers on his tongue were completely healed, and the man had nearly regained his ordinary state of health.

I ought to have mentioned that, when the Procurator Fiscal called on me, he informed me that he had ascertained that a drachm of the *powder* of cantharides, and not the tincture, had been given to Middleton. It was fortunate for the poor fellow that, soon after taking the mixture, he had vomited, which no doubt saved his stomach and bowels, in a great measure, from the influence of so powerful a dose. The state of his mouth and throat is thus also accounted for, by the probable adhesion of particles of the powder to these parts, in the act of rejection.

I now come to the trial of the prisoners, which took place at Aberdeen on the 21st of last month. The facts as above detailed were stated by me in evidence before the Court; and, being particularly questioned as to whether I considered Middleton's life in danger at the time of my first visit to him, I answered that, though I had hopes of his recovery, I could not feel myself justified in certifying him to be free from danger. Being further interrogated respecting the internal administration of cantharides as a medicine, I stated that I could not speak of it from experience, having never ventured to use it in my own practice; that I considered it at all times violent in its operation; and that, as far as I could learn from reading, it had never been given to a greater extent than a few grains at a time, and even that after the stomach had been gradually accustomed to its exhibition. A cross-examination was attempted by a young gentleman acting as counsel for the defence, but without producing any particular alteration in the complexion of his clients' case.

On my examination being concluded, Dr. Dyce, a well-known professor and practitioner of midwifery in Aberdeen, was called on by the above-mentioned counsel, and stated that, from my written report, he should not have considered the man in any danger; adding, at the same time, that he would have been better able to judge if he had seen the patient. He moreover stated that he had, in the course of his own practice, given as much as *ten* grains of the powder of cantharides as a single dose to a patient.

It may be proper to notice that, owing to particular circumstances, only one man was convicted; and he, not being supposed the principal in this shameful business, was only sentenced to two months' confinement in the jail of Aberdeen.

In concluding this hasty, but I trust correct, account of the case, I have to express my regret that medical men should ever be opposed to one another in their evidence given in courts of justice; a difference of opinion tending to expose the most useful and honourable of all professions to the sneers of prejudice and ignorance, and having the effect of lessening medical science in the esteem and confidence of the public.

*London; October 7th,* 1825.

